# Mutant *kri1l* causes abnormal retinal development via cell cycle arrest and apoptosis induction

**DOI:** 10.1038/s41420-024-02022-2

**Published:** 2024-05-24

**Authors:** Rong Zhang, Jiajun Sun, Yabin Xie, Wei Zhu, Meitong Tao, Yu Chen, Wei Xie, Rengui Bade, Shuyuan Jiang, Xiaolei Liu, Guo Shao, Weijun Pan, Chengjiang Zhou, Xiaoe Jia

**Affiliations:** 1 Department of Basic Medicine and Forensic Medicine, Baotou Medical College, Inner Mongolia, Baotou, China; 2 Inner Mongolia Key laboratory of Hypoxic Translational Medicine, Baotou Medical College, Inner Mongolia, Baotou, China; 3 Fourth Hospital of Baotou, Inner Mongolia, Baotou, China; 4 Beijing Key Laboratory of Hypoxic Conditioning Translational Medicine, Xuanwu Hospital, Capital Medical University, Beijing, China; 5 School of Pharmacy, Baotou Medical College, Inner Mongolia, Baotou, China; 6 Center for Translational Medicine and Department of Laboratory Medicine, The Third People’s Hospital of Longgang District, Shenzhen, China; 7 Shanghai Institute of Nutrition and Health, Chinese Academy of Sciences, Shanghai, China

**Keywords:** Differentiation, Neurogenesis

## Abstract

Damage to the ribosome or an imbalance in protein biosynthesis can lead to some human diseases, such as diabetic retinopathy (DR) and other eye diseases. Here, we reported that the *kri1l* gene was responsible for retinal development. The *kri1l* gene encodes an essential component of the rRNA small subunit processome. The retinal structure was disrupted in *kri1l* mutants, which resulted in small eyes. The boundaries of each layer of cells in the retina were blurred, and each layer of cells was narrowed and decreased. The photoreceptor cells and Müller glia cells almost disappeared in *kri1l* mutants. The lack of photoreceptor cells caused a fear of light response. The development of the retina started without abnormalities, and the abnormalities began two days after fertilization. In the *kri1l* mutant, retinal cell differentiation was defective, resulting in the disappearance of cone cells and Müller cells. The proliferation of retinal cells was increased, while apoptosis was also enhanced in *kri1l* mutants. γ-H2AX upregulation indicated the accumulation of DNA damage, which resulted in cell cycle arrest and apoptosis. The *kri1l* mutation reduced the expression of some opsin genes and key retinal genes, which are also essential for retinal development.

## Introduction

In the vertebrate central nervous system (CNS), neural retina neurogenesis is a very good system because the retinal structure and developmental mechanisms are highly conserved [1]. Zebrafish (*Danio rerio*) is a type of spinal model organism. At 72 hpf (hours post fertilization, hpf), the retina of zebrafish is close to maturity, and the retinal structure and function are similar to those of the human retina. The mature zebrafish retina is composed of three separate nuclear layers by two reticular layers. From the outside to the inside, they are the outer nuclear layer, the outer reticular layer, the inner nuclear layer, the inner reticular layer and the ganglion cell layer, and photoreceptor cell bodies exist in the outer nuclear layer. Amacrine glial cells, horizontal glial cells, and Müller glial cells occupy the inner nuclear layer. Ganglion cells exist in the ganglion cell layer, and synapsis between these nuclear layers occurs at the plexiform layers [2].

The ribosome is a highly conserved ribonucleoprotein complex that is the main center of mRNA and protein quality control [3]. Ribosomes are important and highly complex machines responsible for the synthesis of proteins in all cell growth processes, and diseases caused by ribosome biosynthesis disorders are called ribosomal diseases, such as diabetic retinopathy (DR), glaucoma, cataracts and other eye diseases [4–7]. In addition, ribosome biogenesis must respond rapidly to environmental cues mediated by internal and cell surface receptors or stress (oxidative stress, DNA damage, amino acid depletion, etc.) [8]. Impaired ribosomal biosynthesis can cause DNA damage [9]. DNA damage may be caused by various endogenous or exogenous stresses, including oxidative stress, telomere erosion, carcinogenic mutation, genotoxic stress, and metabolic stress.

This study reported that *kri1l* was essential for retinal development. *Kri1l* is an important part of the ribosomal 45 S rRNA cleavage complex and is responsible for the cleavage of ribosomal 18 S rRNA [10]. Kri1l deletion causes disordered ribosome biosynthesis through the accumulation of DNA damage, resulting in cell cycle arrest and apoptosis. At the same time, the *kri1l* mutation reduced the expression of certain opsin genes and key retinal genes.

## Results

### Mutant *kri1l* causes retinal morphological defects

**Fig. 1:**
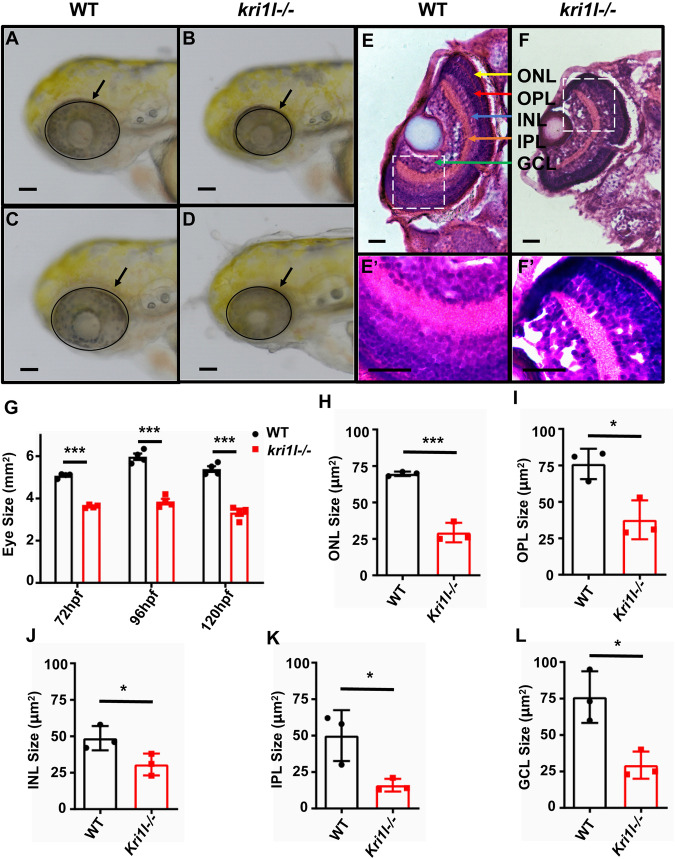
The retinal structure was disrupted in *kri1l−/−* mutants. **A**–**D** Light microscope image of zebrafish embryos at 4 dpf. Scale bar, 100 μm. **G** Statistical analysis of the eye surface area in sibling and *kri1l*−/− mutants. Error bars represent the standard deviation (SD). ***, *P* < 0.001. The black arrows indicate the eye. **E**, **F** H&E staining of frozen embryo eye sections at 4 dpf. Scale bar, 50 μm. The zoom section had shown in **E**′–**F**′. The yellow arrow indicated the ONL (outer nuclear layer). The red arrow indicated the OPL (Outer plaxiform layer). The blue arrow indicated the INL (inner nuclear layer). The orange arrow indicated the IPL (Inner plaxiform layer). The green arrow indicated the GCL (ganglion cell layer). **H**–**L** Statistical analysis of the indicated area in sibling and *kri1l*−/− mutants.

The *kri1l*−/− mutant (*kri1l*^*cas002*^) was obtained from a large-scale forward genetics screen with ENU-mutagenized in zebrafish. And we carried out positional cloning and found a mutantion in *kri1l* (also named *kri1*) gene responsible for the phenotype [11]. In eukaryotic cells, a ribonucleoprotein (RNP) called small subunit (SSU) processome takes charge of generating mature 18 S rRNA and assembling small ribosomal subunit. *Kri1l* is a component of the SSU complex, loss of Kri1l results in instability of 18 S rRNA precursor and dramatic reducation of mature 18 S rRNA. *Kri1l* is essential for the formation of polysome and 40 S ribosome subunits. The retina of zebrafish is close to maturity at 72 hpf, so 72 hpf after fertilization is the best time to observe retinal development. Under an optical microscope, we found that the *kri1l* mutation caused the mutant eyes to decrease compared to wild-type embryos (Fig. 1A–D). After measuring the eye area of *kri1l* mutants at 3 dpf, 4 dpf, and 5 dpf, it was found that the eye area of the *kri1l* mutant was smaller than that of the wild-type embryo (Fig. 1G). To further explore changes in the retina, cryosections of the eyes were subjected to hematoxylin-eosin staining (HE). The results showed that the boundaries of each retinal cell layer became fuzzy, and each layer cell became narrower in the *kri1l* mutant, indicating that the cell number was reduced in each retinal layer (Fig. 1E, F). We counted the area of each layer cells in the retina, and the total area in each layer significantly decreased (Fig. 1H–L). Hoechst staining also indicated a significant decrease in the number of cells in each layer. These data indicated the retinal development was severely disrupted in *kri1l*−/−.

After immunofluorescence staining and photographing, all embryos were extracted for genomic DNA and genotyped by sequencing. There was a 38 bp deletion in the cDNA of *kri1l* gene in mutants (Fig. S1A). Genomic DNA sequencing of *kri1l* gene revealed that a consensus splicing donor site at the boundary between exon1 and intron1 was disrupted by a T-to-G transitionin in mutants (Fig. S1B). This transition T-to-G spliced from the earlier GT position in exon1 and yielded an alternative splicing transcript with a frame shift (Fig. S1C). We designed primers (which located on exon 1 and exon 2), and amplified from genomic DNA, and found that the product in the mutants was 192 bp, significantly smaller than that in wild-type 230 bp (Fig. S1D).

### *kri1l* is required for retinal differentiation

**Fig. 2:**
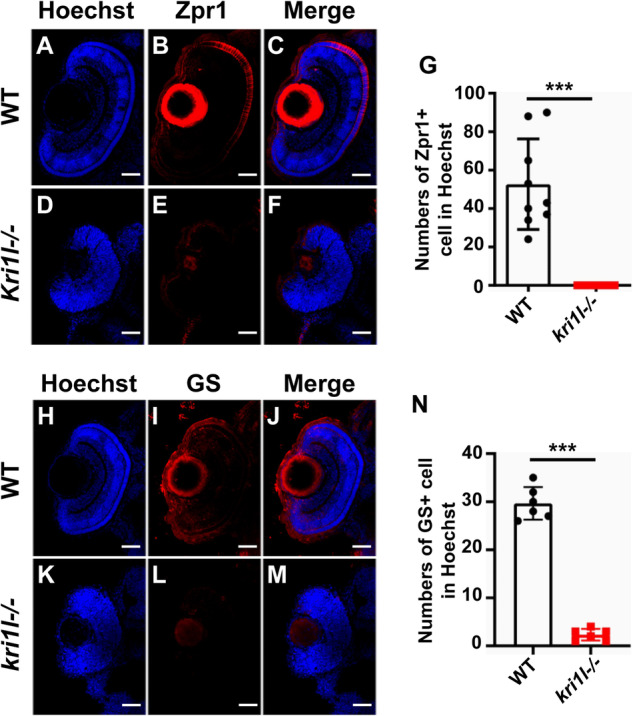
Photoreceptor cells and Müller glial cells were absent. **A**–**F** Representative Zpr-1 (for photoreceptor cells) immunofluorescence images for frozen embryo eye sections from sibling **A**–**C** and *kri1l−/−* mutants **D**–**F** at 4 dpf. **G** Statistical analysis of Zpr-1+ cells in sibling and *kri1l*−/− mutants. **H**–**M** Representative GS (for Müller glial cells) immunofluorescence images for frozen embryo eye sections from sibling **H**–**J** and *kri1l*−/− mutants **K**–**M** at 4 dpf. **N** Statistical analysis of Müller glial cells in sibling and *kri1l*−/− mutants. Error bars represent the standard deviation (SD). *, *P* < 0.05; **, *P* < 0.01; ***, *P* < 0.001. Scale bar, 50 μm.

To determine the differentiation patterns in *kri1l*−/− mutants, we examined differentiated retinal neurons (Zpr-1 for photoreceptor cells [12]) and Müller glial cells (GS, markers for Müller glia cells [13]). We found these differentiated retinal neurons were substantially reduced, including Zpr-1 (Fig. 2A–F) and GS (Fig. 2H–M). The number of Zpr-1+ and GS+ cells was significantly reduced in *kri1l*−/− mutants (Fig. 2G, N).

**Fig. 3:**
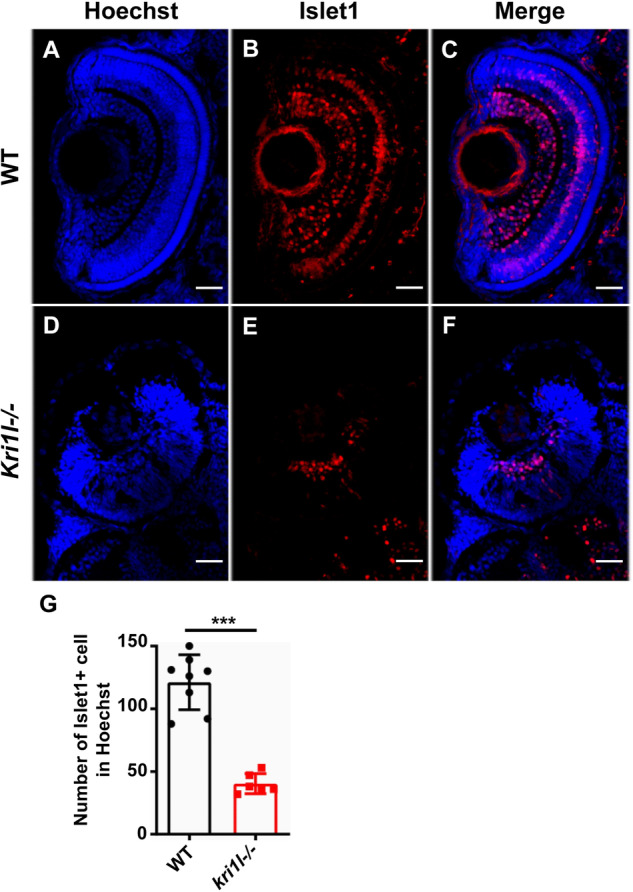
Inner nuclear layer cells was also impaired in *kri1l−/−* mutants. **A**–**F** Representative Islet1 (for inner nuclear layer cells) immunofluorescence images for frozen embryo eye sections from sibling **A**–**C** and *kri1l*−/− mutants **D**–**F** at 4 dpf. **G** Statistical analysis of islets + cells in sibling and *kri1l*−/− mutants. Error bars represent the standard deviation (SD). ***, *P* < 0.001. Scale bar, 50 μm.

Another retinal differentiation marker Islet1 (Islet1 for inner nuclear layer cells), were decreased (Fig. 3A–G). The inner nuclear layer contains the cell body of bipolar cells, horizontal cells, and amacrine cell cells. And the migration of retinal neurons occur from the inner side to the outer side, starting from inner nuclear layer cells. The Islet1 positive cells decreased suggested nearly all differentiated retinal neurons were deficiency.

**Fig. 4:**
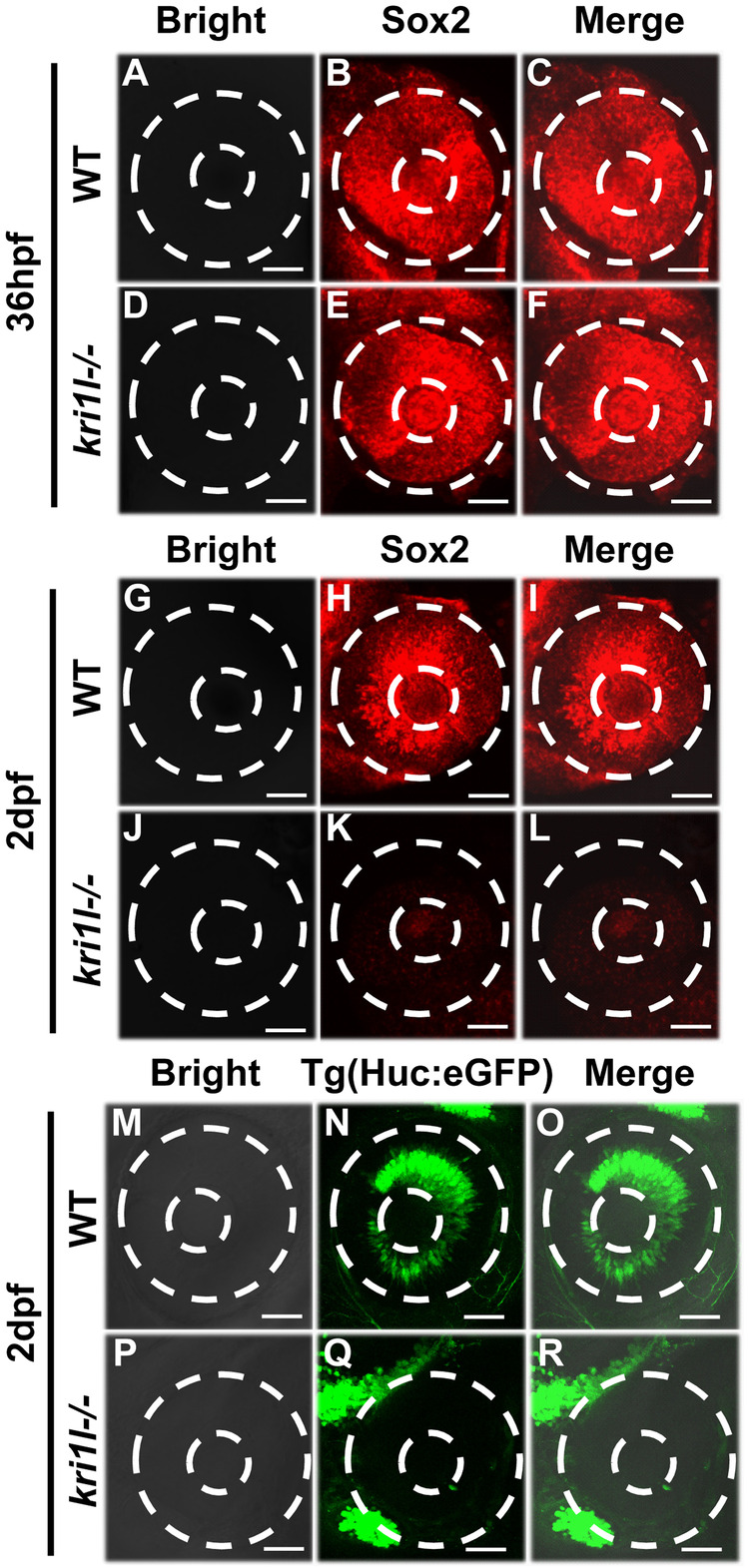
Mutation of *kri1l* impaired RPC differentiation. **A**–**F** The distributions of Sox2+ cells (markers for RPC) in whole-mount retinas from WT and *kri1l*−/− mutants at 36 hpf. **G**–**L** The distributions of Sox2+ cells (markers for RPC) in whole-mount retinas from WT and *kri1l*−/− mutants at 2 dpf. **M**–**R** The distributions of *HuC*:EGFP (postmitotic neurons)-labeled cells in whole-mount retinas from WT and *kri1l*−/− transgenic zebrafish at 2 dpf. The dashed circles show the eyes and lenses. Scale bar, 100 μm.

Retinal lamination is initiated by the migration of postmitotic neurons to the appropriate cell layer. When neurons migrate to the appropriate retinal region, they mature and establish synapses between different cell layers and differentiate into seven types of retinal cells: Müller glia cells, bipolar cells, cone photoreceptors and so on. Multipotent RPCs (retinal progenitor cells) can differentiate into all types of retinal neurons [2, 14]. To understand when the differentiation of retinal cells initiated failure, we traced retinal progenitor cell (sox2, markers for RPCs) expression at sequential developmental time points. *Sox2* expression was normal in the retina at 36 hpf (Fig. 4A–F). The initial decrease in *sox2* expression was detectable in the retinal region at 2 dpf (Fig. 4G–L). To further confirm these results, Tg(*HuC*:eGFP) living embryos were observed at 2 dpf, which labeled neurons. Consistently, *HuC*:eGFP+ cells were dramatically reduced in *kri1l*−/− retinas (Fig. 4M–R). Above all, the retinal differentiation defects initiated from 2dpf, and RPCs could not differentiate into various retinal lineages during neurogenesis in the absence of *kri1l*.

### Defects in photoreceptor cells in the retina caused a fear of light response in *kri1l* mutants

**Fig. 5:**
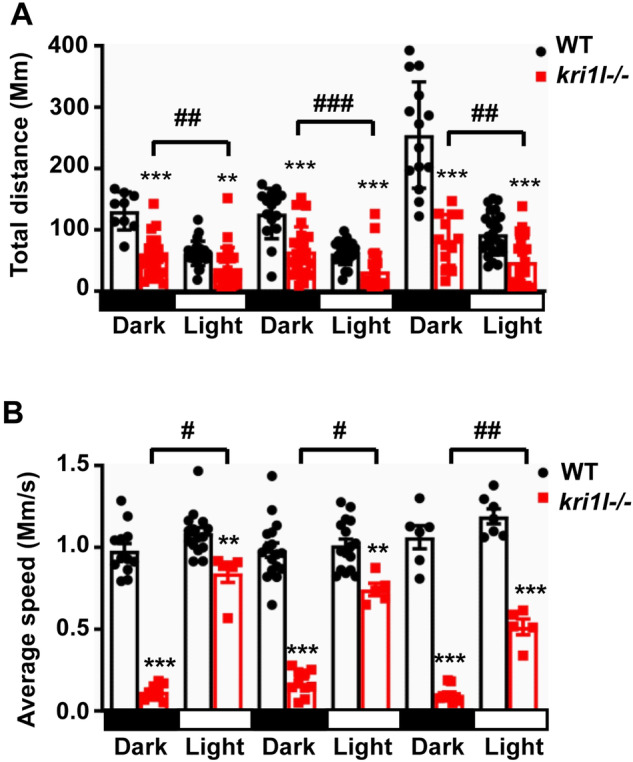
Effects of *kri1l* mutation on the response to light stimulus. **A** The total distance of the larvae during the 5 min dark and 5 min light period. **B** The average swimming speed of the larvae during the 5 min dark and 5 min light period. * are compared between WT and *kri1l*−/− mutants, **, *P* < 0.01; ***, *P* < 0.001; # are compared between dark period and light period in *kri1l*−/− mutants, #, *P* < 0.05; ##, *P* < 0.01; Results are presented as the mean ± SD.

To further explore the response of the *kri1l* mutant to light stimulus in the absence of photoreceptor cells, we performed alternating light and dark light cycles to stimulate equal behavioral experiments. The total distance and swimming speed of *kri1l*−/− were all lower than those of wild-type embryos (Fig. 5A). The total distance of the *kri1l* mutant in the dark was less than that in the light (Fig. 5A). The *kri1l* mutant showed an increase in average speed and startle within 5 min of light. After turning off the light source, the speed quickly decreased, but the average speed of wild-type embryos remained basically unchanged under different light conditions (Fig. 5B). These data suggested that the swimming speed of mutant larvae exhibited an uptick in the light period, indicating that the mutant larvae showed a fear of light response.

### Proliferation of retinal cells increased and apoptosis was also enhanced in *kri1l* mutants

**Fig. 6:**
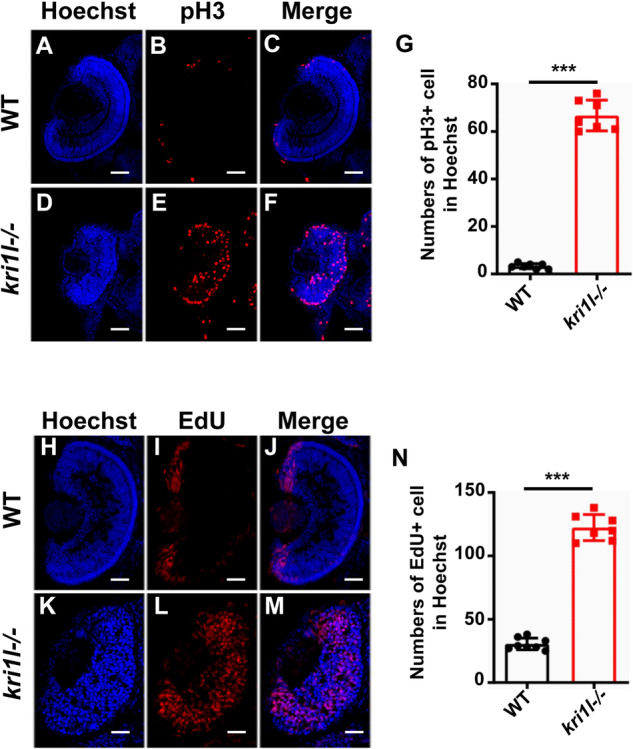
Proliferation of retinal cells increased in *kri1l−/−* mutants. **A**–**F** pH3 (marked for G2/M-phase cells) staining in frozen embryo eye sections of sibling **A**–**C** and *kri1l−/−* mutants **D**–**F** at 4 dpf. **G** The pH3+ cell quantitative results are summarized in **A**–**F**. **H**–**M** EdU (marked for S-phase cells) staining in frozen embryo eye sections of sibling **H**–**J** and *kri1l−/−* mutants **K**–**M** at 4 dpf. **N** The EdU+ cell quantitative results are summarized in **H**–**M**. Error bars represent the standard deviation (SD). ***P ≤ 0.001 (Student’s t test). Scale bars, 50 μm.

To observe the mechanism of the decrease in retinal cells, we performed cell proliferation and apoptosis in retinas by pH3 and EdU immunofluorescence and TUNEL assays. pH3 (phosphorylation at Ser 10 of histone H3) immunofluorescence and EdU staining were used to detect cell proliferation. pH3 staining was enhanced, which can mark cell proliferation in the G2/M division stage [15] (Fig. 6A–G). EdU labeling also increased, which was used to detect cell proliferation in the S stage [16] (Fig. 6H–N). The pH3 and EdU staining all indicated the cell proliferation was elevated in mutants.

**Fig. 7:**
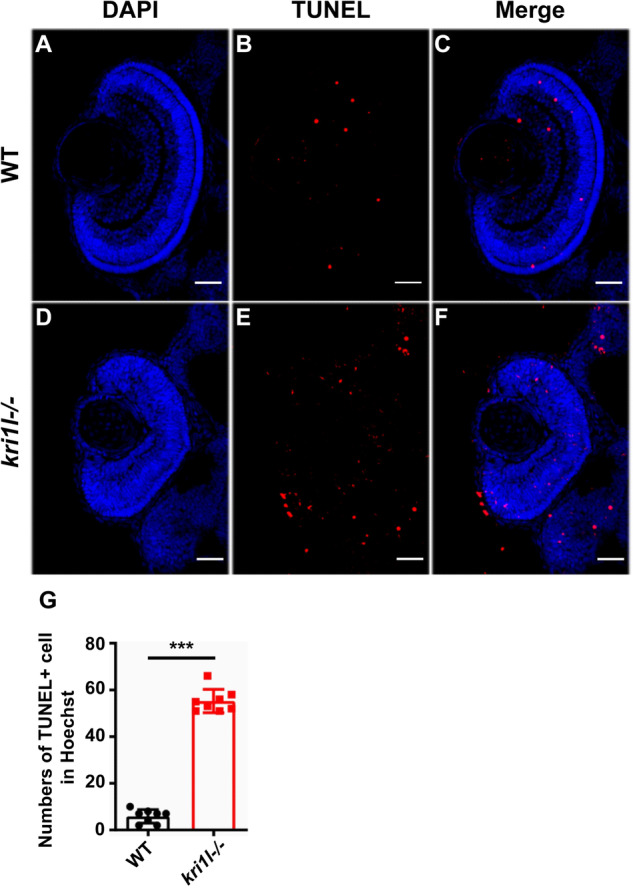
The apoptosis pathway was activated in *kri1l−/−* mutants. **A**–**F** TUNEL staining in frozen embryo eye sections of sibling **A**–**C** and *kri1l−/−* mutants **D**–**F** at 4 dpf. **G** Quantitative results are summarized. Error bars represent the standard deviation (SD). ***, *P* ≤ 0.001 (Student’s t test). Scale bars, 50 μm.

**Fig. 8:**
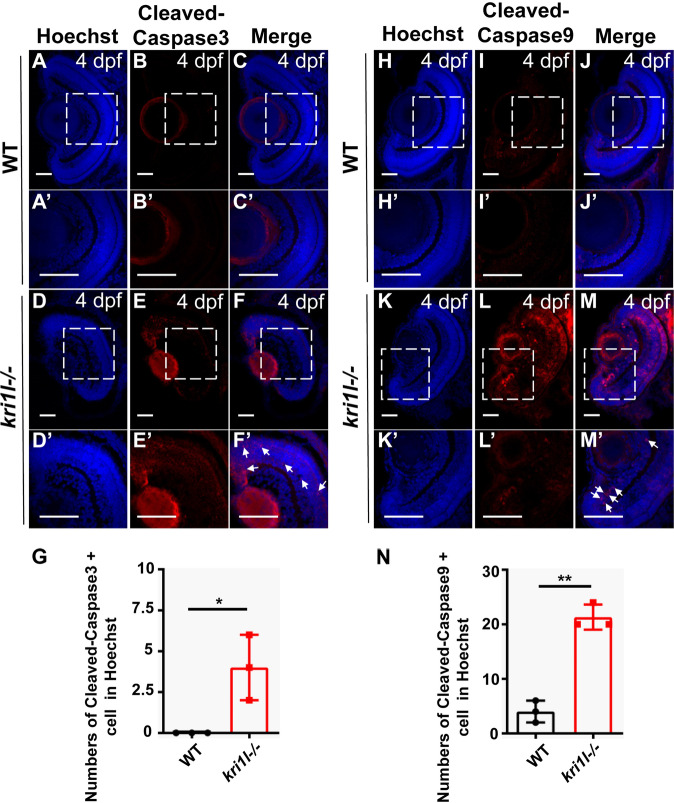
Another apoptosis marker caspase 3 and caspase 9 was activated in *kri1l−/−* mutants. **A**–**F** Representative cleaved caspase3 immunofluorescence images for frozen embryo eye sections from sibling **A**–**C** and *kri1l−/−* mutants **D**–**F** at 4 dpf. The zoom section had shown in **A**′-**F**′. **G** Statistical analysis of cleaved caspase3 + cells in sibling and *kri1l−/−* mutants. **H**–**M** Representative cleaved caspase9 immunofluorescence images for frozen embryo eye sections from sibling **H**–**J** and *kri1l−/−* mutants **K**–**M** at 4 dpf. The zoom section had shown in **H**′–**M**′. **N** Statistical analysis of cleaved caspase9 + in sibling and *kri1l−/−* mutants. Error bars represent the standard deviation (SD). *, *P* < 0.05; **, *P* < 0.01; ***, *P* < 0.001. Scale bar, 50 μm.

TUNEL staining detects cell apoptosis [17, 18]. The results showed that the TUNEL-positive signal was noticeably elevated in the *kri1l* mutant, suggesting the activation of apoptosis (Fig. 7A–G). To define the apoptosis, we looked at additional markers of apoptosis such as cleaved caspase 3 and cleaved caspase 9. The number of cleaved caspase 3 + cells (Fig. 8A–G) and cleaved caspase 9 + cells (Fig. 8H–N) in *kri1l*−/− mutants were significantly increased. Together with TUNEL results (Fig. 7), all these results suggested that the apoptosis level was elevated in *kri1l*−/− mutants.

### Accumulation of DNA damage resulted in cell cycle arrest and apoptosis

**Fig. 9:**
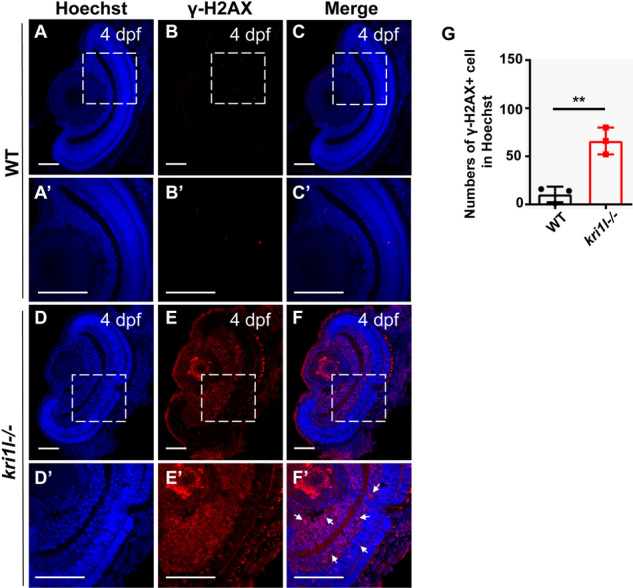
Accumulation of DNA damage in *kri1l−/−* mutants. **A**–**F** Immunofluorescence analysis using the anti-γ-H2AX antibody in sibling **A**–**C** and *kri1l−/−* mutant **D**–**F** retinas at 4 dpf. The zoom section had shown in **A**′–**F**′. **G** Statistical analysis of γ-H2AX + cells in sibling and *kri1l−/−* mutants. Scale bar, 50 μm.

Many studies have shown that DNA damage affects cell proliferation and apoptosis, hindering cell cycle progression [19–21]. We suspected that there was DNA damage in the *kri1l* mutant. To test this hypothesis, we performed DNA damage detection in the *kri1l* mutant. γ-H2AX is phosphorylated H2AX, and the phosphorylation site is located at serine 139. The expression of the γ-H2AX gene can detect DNA break sites [22, 23]. The results showed that the γ-H2AX signal (Fig. 9A–G) was enhanced in the retinas of the *kri1l* mutant.

### The expression of some opsin genes and key retinal genes was downregulated in *kri1l* mutants

**Fig. 10:**
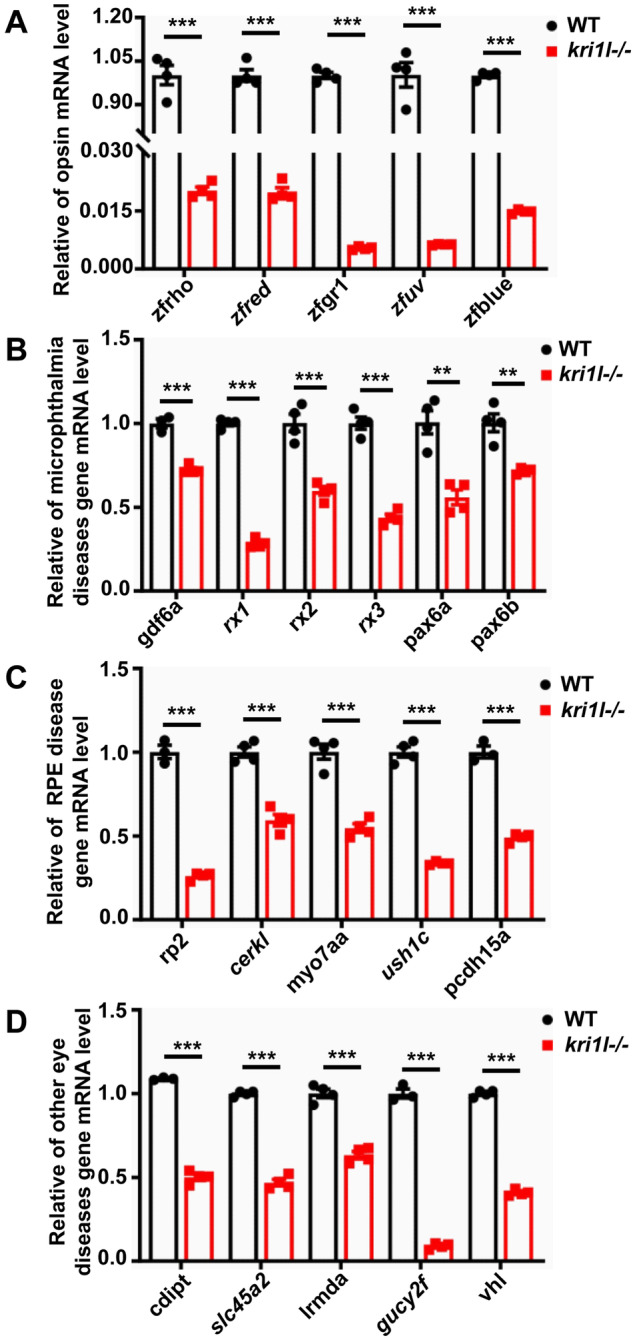
The expression of some opsin genes and key retinal genes was downregulated in *kri1l* mutants. The relative expression of in sibling and *kri1l−/−* mutant embryos at 4 dpf. **A** Relative expression of opsin gene. **B** Relative expression of microphthalmia diseases gene. **C** Relative of RPE disease gene mRNA level. **D** Relative of other eye diseases gene mRNA level. Error bars represent standard deviation (SD). **P ≤ 0.01; ***P ≤ 0.001.

The mRNA levels of five opsin genes and some key retinal genes were also examined by qRT‒PCR tests in the *kri1l* mutant. The mRNA levels of the zfrho, zfred, zfgr1, zfuv and zfblue genes, which encode rhodopsin, red, green, ultraviolet and blue opsins, respectively, were significantly and consistently downregulated. The gdf6a, rx1, rx2, rx3, pax6a and pax6b genes are related to microphthalmia. The rp2, cerkl, myo7aa, ush1c and pcdh15a genes are related to retinitis pigmentosa. The cdipt gene is related to cataracts. The slc45a2 and lrmda genes are associated with albinism [2]. The gucy2f gene is related to Leber’s congenital amaurosis (LCA). The vhl gene is related to the development of retinal blood vessels. The results showed that the mRNA levels of the opsin genes were reduced in the *kri1l* mutant (Fig. 10A). The mRNA expression levels of genes related to microphthalmia (Fig. 10B), retinitis pigmentosa (Fig. 10C), cataract (Fig. 10D), ocular albinism (Fig. 10D), Leber’s congenital amaurosis (Fig. 10D), and retinal vascular development (Fig. 10D) were all downregulated in the *kri1l* mutant. Therefore, the downregulation of expression of some visual protein genes and key retinal genes in the kri1l mutant may lead to retinal dysplasia.

## Discussion

This study showed that the *kri1l* mutation caused abnormal retinal development, with fuzzy boundary retinal cell layers, and each layer cell became narrower and smaller in the *kri1l* mutant. Retinal differentiation was damaged because photoreceptor cells and Müller glial cells almost disappeared in *kri1l* mutants. The lack of photoreceptor cells caused a fear of light response. It might be the accumulation of DNA damage that resulted in cell cycle arrest and apoptosis. At the same time, the *kri1l* mutation reduced the expression of certain opsin genes and key retinal genes. The working model for Kri1l dysfunction abnormal retinal development was in Fig. S2.

Kri1l gene research first appeared in 2000 and is involved in the splicing process of 18 S rRNA, and yeast mutant strains that lack the kri1 gene exhibit slow growth [10, 24]. Subsequent reports mentioned that loss of *kri1l* caused ribosomal biogenesis defects, accumulation of misfolded proteins and activation of PERK-eif2a signaling. These deficiencies subsequently hyperactivate autophagy and ultimately lead to the inhibition of HSPC proliferation [11]. In addition, all embryos homozygous for the *kri1l* mutation died within 5–10 days of fertilization. The evidence could attest to the importance of *kri1l* development. In human diseases, the KRI1 mutation may be relevant with severe iron deficiency anemia [25]. And KRI1 were significantly correlated with esophageal carcinoma tumor location, lymph node metastasis, and age of patients. KRI1 had the highest mutation frequency from the TCGA database [26].

Our results suggested that when *kri1l* was mutated, the structure of the retina was destroyed, leading to the appearance of ommatidia and fuzzy boundary retinal cell layers, and each layer cell became narrower and smaller. Studies have reported that plk1, vps28, psmd2, ran, sec13 [27], ccdc94 [28], and tln1 [29] mutations cause ommatidia in zebrafish embryos. The plk1 mutant had abnormal retinal development and did not form a normal retinal morphology. The vps28 mutant had slightly smaller eyes, but the retinal stratification was normal; the psmd2 and ran mutants had damaged retinal stratification [30]. These retinal defects were very similar to our results (Fig. 1).

Photoreceptor cells (cones and rods) are used to receive light signals, process the signals and transmit them to other retinal nerve cells. When the retina is lost, Müller cells re-enter the cell cycle to proliferate and differentiate into damaged nerve cells, thereby repairing the damage to the retina [31–33]. Previous studies have reported cone differentiation defects in her9 and lca535 [34] mutants, and Müller cells were reduced and there were developmental disorders in the her9 mutant [35]. Our results showed that the retina of the *kri1l* mutant had almost no cones or Müller cells. This is consistent with our results (Figs. 2 and 3).

Subsequently, to further explore the response of the *kri1l* mutant to light in the absence of photoreceptor cells, we performed alternating light and dark light cycles to stimulate equal behavioral experiments. We found that the *kri1l*−/− mutants moved intensely during light conditions, but the swimming speed decreased during dark conditions. Conversely, there was no significant difference in wild-type embryos. These results suggested a response of fear of light when illuminated when *kri1l* was deleted (Fig. 5B). Cone dysfunction syndrome is a disease of cone dystrophy, with varying degrees of nystagmus and photophobia [36]. Mutations in CNGA3 impair the light-sensing function of cone cells, leading to color blindness, solar blindness, poor vision and photophobia [37]. Based on these studies, we suspected that the photophobic response of the *kri1l* mutant may be related to the differentiation defect of cone cells.

In addition, we found that *kri1l* mutants increased cell proliferation, and apoptosis was also enhanced. To explain this phenomenon, we detected γ-H2AX gene expression in *kri1l* mutants. The results showed that the expression of the γ-H2AX was upregulated, indicating that there was accumulation of DNA damage in the *kri1l* mutant, which in turn induced cell cycle arrest and apoptosis. The latest research reported that Prpf31 (pre-mRNA processing factor 31) mutations caused DNA damage and mitotic abnormalities, leading to TP53-dependent apoptosis [38]. Therefore, we speculate that the *kri1l* mutation caused cell cycle arrest and apoptosis, possibly due to the accumulation of DNA damage.

Color vision comes from different visual pigment proteins (Opsin) expressed by the retinal photoreceptors [39]. In our experiment, due to the mutation of *kri1l*, the levels of opsin mRNA in the retina decreased, which might be caused by defective photoreceptor cell differentiation. The gdf6a, rx1, rx2, rx3, pax6a, and pax6b genes are related to microphthalmia. The rp2, cerkl, myo7aa, ush1c, and pcdh15a genes are related to retinitis pigmentosa. The cdipt gene is related to cataracts. The slc45a2 and lrmda genes are associated with albinism. The gucy2f gene is related to Leber’s congenital amaurosis. The vhl gene is related to the development of retinal blood vessels [2]. These genes are not only related to eye diseases but are also key genes for retinal development. Mutations in the *kri1l* gene resulted in lower mRNA levels of these genes, indicating that these genes are essential for the development of the retina.

In conclusion, *kri1l* mutation can destroy the development of the retina by inducing cell cycle arrest and apoptosis, indicating that *kri1l* is very important for the development of the retina. This study revealed the pathogenesis of retinal development caused by mutations in ribosome-related proteins and provided experimental data for retinal-related diseases.

## Materials and methods

### Zebrafish husbandry and maintenance

The *kri1l* +/- zebrafish line, with 40 individuals at 6 months old, half female and half male, was cultured in the zebrafish model animal platform of Baotou Medical College. Tg(*HuC*:eGFP) (CZ160) was purchased from the China Zebrafish Resource Center. The zebrafish were maintained in a circulating filtration system with pH: 7.0 ± 1.0; temperature: 28 ± 1 °C; conductivity: 400–500 S/cm; and a 12 h:12 h cycle of day and night. Zebrafish were fed live brine shrimp (*Artemia salina*) twice daily. The night before the experiment began, male and female fish were placed in the same hatching box and separated by a comb. The next morning, the zebrafish began to spawn at the moment of light. Embryos were collected with 0.005% 1-phenyl-2-thiourea (PTU) E3 medium (egg water). PTU solution can inhibit pigmentation. Mutants *kri1l*−/− were morphologically indistinguishable from wild-type siblings before 3 dpf with normal blood flow and heart beating. For 36hpf to 3dpf, the embryos were collected for experiments. After immunofluorescence staining and photographing, all embryos were extracted for genomic DNA and genotyped by sequencing. For 4dpf, embryos can be distinguishable by small head, small eyes and cardiac edema.

### Ethics statement

All animals and experiments were reviewed and approved by the Experimental Animal Ethics Committee of Baotou Medical College, Inner Mongolia University of Science and Technology.

### Morphological analysis

Zebrafish embryos were anesthetized with tricaine at a volume fraction of 0.08%, and then the embryos were placed on 3% methylcellulose and photographed with a stereo microscope.

### Frozen section

Embryos at 72 hpf were fixed with 4% PFA overnight at 4 °C and then dehydrated overnight. The heads were used for frozen sectioning, while the tails were used for genotyping. Next, the permeated embryos were embedded in Tissue-Tek® O.C.T. compound (Sakura Finetek, USA) and were frozen at -80 °C for more than 6 h. Cryosections were collected at 6–8 μm in thickness with frozen microtomy (Leica). The sections were stored for H&E staining, immunofluorescence, and TUNEL assays.

### Histologic analysis

Briefly, the collected cryosections were fixed in 4% paraformaldehyde for 10 min and washed in water for 2 min. The cryosections were stained with filtered hematoxylin solution (Beyotime, C0105) for 8 min at room temperature (RT). Following a wash in distilled water for 2 min, the sections were treated with 0.3% hydrochloric acid ethanol solution for 2 s, washed with water for 2 min, counterstained in eosin dye for approximately 30 s at RT, placed in 70%, 95%, and 100% ethanol dehydrate in the solution for 10 s, and mounted on slides with antifade mounting medium. Finally, high-resolution images of the H&E-stained sections were obtained under a microscope (Nikon, SMZ18). *N* ≥ 6, the experiment was repeated at least three times.

### Locomotion analysis in larval zebrafish

Different groups of zebrafish embryos were placed into a 24-well plate, and the zebrafish embryo movement behavior instrument was controlled to collect 30 min of motion video. Among them, there was no light for the first 5 min, and there was light for 5 min. The light and dark cycles were repeated 3 times. EthoVision XT software was used to export motion speed parameters. *N* ≥ 24, the experiment was repeated at least three times.

### Terminal deoxynucleotidyl transferase dUTP Nick End Labeling (TUNEL) staining

The cryosections were stained using an in situ cell death detection kit. The cryosections were circled with a hydrophobic pen, fixed in 4% paraformaldehyde for 20 min, treated with acetone at -20 °C for 7 min and 1% sodium citrate in PBS for 1 h, stained with a TUNEL staining kit, and stained with 0.1% Tween’s PBS to terminate the reaction. Anti-quenching reagent was used to seal the coverslip. *N* ≥ 6, the experiment was repeated at least three times.

### Immunofluorescence

The cryosections were treated with acetone at -20 °C for 7 min. Samples were blocked with blocking buffer (10% goat serum in PBS with 0.5% Triton X-100) for 2 h at room temperature and incubated with primary antibody overnight. Primary antibodies included Zpr1 (abcam, ab174435, 1:300), GS (Merck, MAB302, 1:300), phosphorylated histone H3 (CST, 3377, 1:500), γ-H2AX (abcam, ab11174, 1:300), Istel1 (GeneTex, GTX102807, 1:200), caspase 3 (CST, #9661, 1:300), and caspase 9 (abcam, ab202068, 1:500). Secondary antibodies conjugated with Alexa Fluor488 were used at a 1:500 dilution. The reaction was terminated with 0.1% Tween in PBS. Hoechst (Beyotime, C1022) dye was used to stain the nucleus (1:2500), and the slides were mounted with antifade mounting medium. Images were obtained with a confocal microscope (Nikon, A1+ Confocal Microscope). *N* ≥ 6, the experiment was repeated at least three times.

### Detection of cell proliferation by EdU staining

The embryos were treated with 2 mM 5-ethynyl-2-deoxyuridine (EdU) for 20 min at 4 °C and then washed 3 times with egg water. The embryos were transferred to egg water in an incubator at 28.5 °C. After 4 h, the embryos were fixed in 4% paraformaldehyde overnight, dehydrated in a methanol gradient overnight and rehydrated. Zebrafish embryos were used for frozen sectioning, and then the Click-iT Plus EdU Imaging Kit (Invitrogen, C10640) was used to process the sections for 30 min. The reaction was terminated using PBS with 0.1% Tween. N ≥ 6, the experiment was repeated at least three times.

### Live imaging of Tg(*HuC*:eGFP) embryos

The Tg(*HuC*:eGFP) live embryos were anesthetized with 0.08% tricaine and mounted in 0.1% low melting point agarose for imaging with a Nikon A1+ confocal microscope (under a 20× water-immersion objective). *N* ≥ 24, the experiment was repeated at least three times.

### Whole mount in situ hybridization

To detect sox2 mRNA, embryos were first hybridized with the DIG-labeled antisense *sox2* RNA probe, incubated at 4 °C overnight with a peroxidase (POD)-conjugated anti-DIG antibody (1:500; Roche), and stained with Alexa Fluor cy3-conjugated tyramide as substrate (PerkinElmer). Images were obtained with a confocal microscope (Nikon, A1+ Confocal Microscope). The experiment was repeated at least three times. N ≥ 24, the experiment was repeated at least three times.

### Quantitative real-time polymerase chain reaction (qRT‒PCR)

Total RNA was extracted from 16 zebrafish embryos using Trizol reagent. Reverse transcription was performed with the Thermo Scientific RNA Reverse Transcription Kit. 2× PCR Mix (TaKaRa, Premix Ex Taq) containing SYBR Green I was used for the real-time quantitative PCR analysis with the Roche Applied Science Fast Real-Time PCR System. The corresponding gene primers are shown in Table S1. The experiment was repeated at least three times.

### Statistical analyses

GraphPad Prism 8 software was used to perform t tests for all statistical analyses. *P* < 0.05 indicated a statistically significant difference, and all the values are the mean ± standard deviation. Each experiment was repeated at least three times.

### Supplementary information

Table S1

Supporting Information Legends

Figure S1

Figure S2

## Data Availability

The data that support the findings of this study are available from the corresponding author upon reasonable request.
