# Supplementary material for: Mutant kri1l causes abnormal retinal development via cell cycle arrest and apoptosis induction
Source: Cell Death Discov. 2024 May 24;10:251. doi: 10.1038/s41420-024-02022-2 (PMC11126728; doi:10.1038/s41420-024-02022-2)
Supplement: Supplementary file 2 — Supporting Information Legends [file 41420_2024_2022_MOESM2_ESM.docx]

**Supporting Information Legends**

**Figure S1. Confirmation of the change in expression of Kri1l.** (A) The sequencing result of *kri1l* cDNA from mutants showed a 38bp deletion comparing to *kri1l* cDNA from wild-type siblings. (B) The sequencing result of *kri1l* genomic DNA showed a T-G transitionat the *kri1l* exon 1-intron 1 consensus splicing donor site. (C) We designed primers (which located on exon 1 and exon 2), and amplified from genomic DNA, and found that the product in the mutants was 192bp, significantly smaller than that in wild-type 230bp (Figure S1D).

**Figure S2. Working model for Kri1l dysfunction abnormal retinal development.**

**Supplemental Table 1**

All primers used for this study are provided in Supplemental Table.
