# Supplementary figures and images for: Mutant kri1l causes abnormal retinal development via cell cycle arrest and apoptosis induction
Source: Cell Death Discov. 2024 May 24;10:251. doi: 10.1038/s41420-024-02022-2 (PMC11126728; doi:10.1038/s41420-024-02022-2)

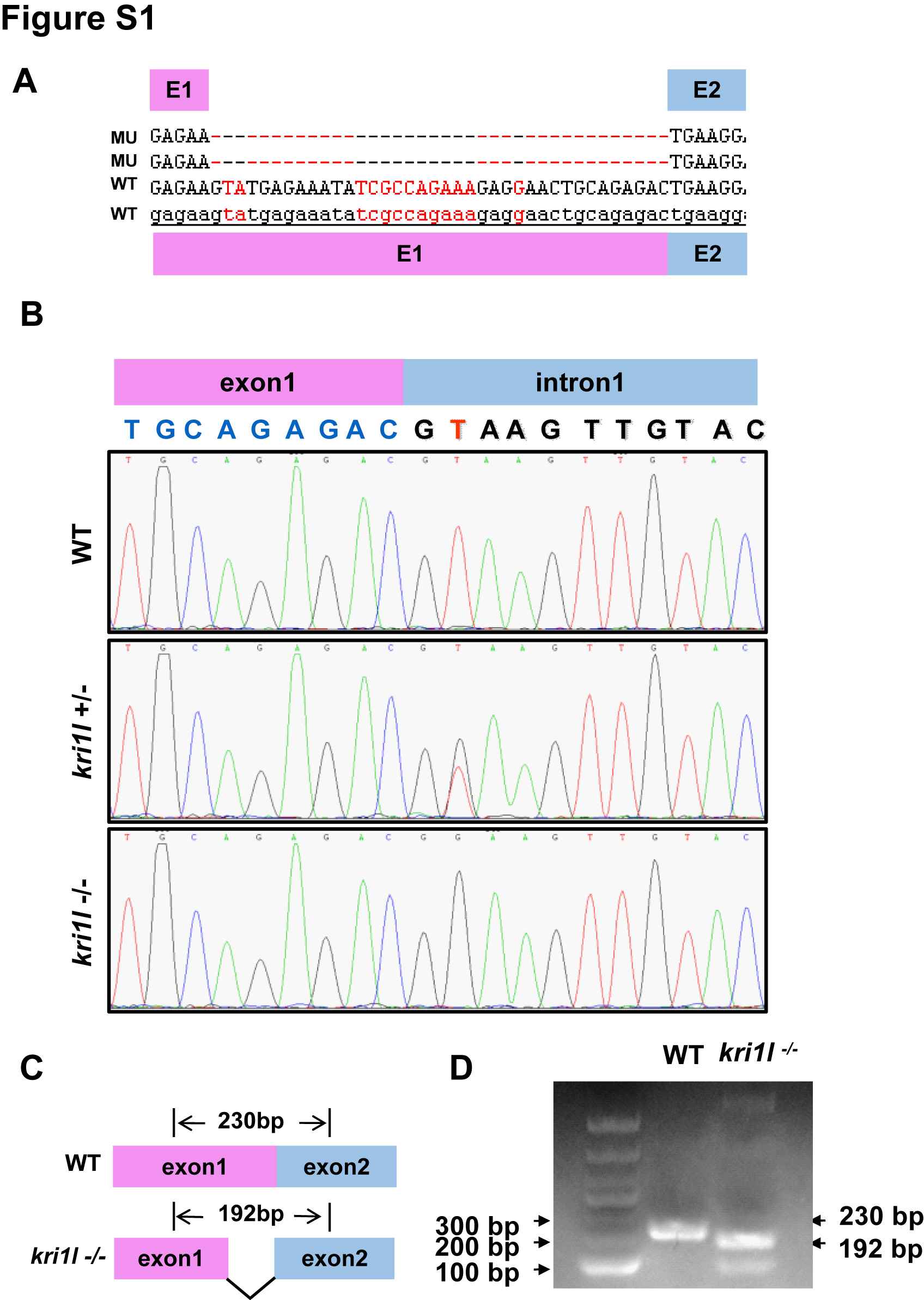

Supplement: Supplementary file 3 — Figure S1 [file 41420_2024_2022_MOESM3_ESM.jpg]

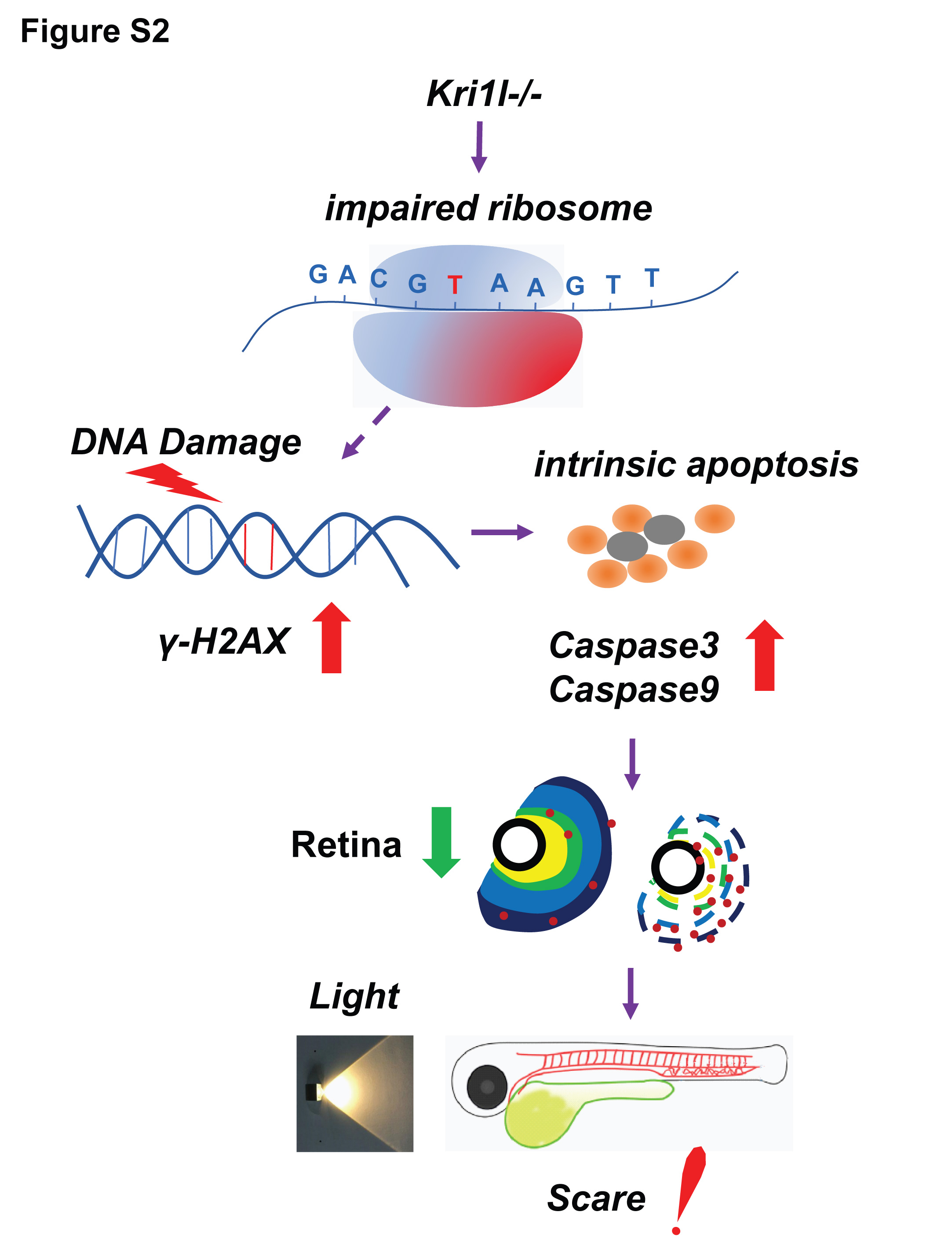

Supplement: Supplementary file 4 — Figure S2 [file 41420_2024_2022_MOESM4_ESM.jpg]
